# An Australasian Survey of Neonatal Clinicians on Clinical Utility of Point‐of‐Care Bowel Ultrasound in Diagnosis of Necrotising Enterocolitis

**DOI:** 10.1002/ajum.70007

**Published:** 2025-06-13

**Authors:** Archana Priyadarshi, Sheryl Rogerson, Nadia Badawi, Stephanie Morakeas, Amy Phu, Mark Tracy

**Affiliations:** ^1^ Neonatal Intensive Care Unit Westmead Hospital Sydney New South Wales Australia; ^2^ The University of Sydney Sydney New South Wales Australia; ^3^ Department of Neonatal Intensive Care Unit The Royal Women’s Hospital Melbourne Victoria Australia; ^4^ Grace Neonatal Intensive Care Unit The Children’s Hospital Westmead Sydney New South Wales Australia

**Keywords:** bowel ultrasound, necrotising enterocolitis, point‐of‐care

## Abstract

**Introduction:**

Necrotising enterocolitis (NEC) is a life‐threatening intestinal disease of the newborn characterised by ischaemia, inflammation and bowel necrosis. Due to the lack of biochemical markers and nonspecific clinical signs in early NEC, bowel ultrasound has gained popularity as a diagnostic tool. This survey aimed to investigate the opinions of neonatal clinicians on the practice of point‐of‐care bowel ultrasound for the diagnosis of NEC.

**Methods:**

This quantitative study utilised a cross‐sectional online single‐invitation survey sent to neonatal clinicians using point‐of‐care ultrasound across Australia, New Zealand and Singapore. We aimed to explore the barriers to, and facilitators of the clinical practice of bowel ultrasound in NEC using the survey responses.

**Results:**

The survey results indicate a clear need for a dedicated training module in Australasia to equip neonatal clinicians with the skills for point‐of‐care bowel ultrasound assessment. Most (95%) of neonatal clinicians practising point‐of‐care ultrasound agreed that performing a bowel ultrasound in the suspected diagnosis of NEC is helpful or may be helpful, with 87% expressing interest in undertaking training.

**Conclusion:**

The study's findings reveal a strong interest among neonatal clinicians in Australia and New Zealand to learn point‐of‐care bowel ultrasound for NEC diagnosis. This interest not only sets the stage for a collaborative approach in planning and developing a training programme but also has the potential to significantly improve NEC diagnosis and patient outcomes in clinical practice.

## 1 Introduction

Necrotising enterocolitis (NEC) is a severe inflammatory condition of the bowel characterised by ischaemia, inflammation and necrosis [1]. This is a progressive disease, often rapid in onset and can lead to life‐long complications such as short‐gut syndrome and death [1, 2, 3]. A plain abdominal radiograph is the gold standard in diagnosing NEC, with pneumatosis intestinalis and portal venous gas as the hallmark signs [4, 5, 6]. However, these signs are not always present, particularly in the early stages of the disease. Furthermore, there is no biomarker for the early identification of NEC [1, 3]. It is thus not uncommon for NEC to be overdiagnosed, particularly in extremely preterm infants who commonly experience abdominal distention and feed intolerance from the use of noninvasive respiratory support [7]. Exploring new means of early recognition is important, as these infants are often critically sick, requiring transfer to a surgical facility for monitoring of the progression of the disease.

Radiologists published state‐of‐the‐art bowel ultrasound findings in NEC almost two decades ago [8, 9, 10, 11], and there has been a recent surge in interest among neonatologists and surgeons in the use of this skill to improve diagnostic outcomes [12, 13]. Neonatal clinicians across tertiary Neonatal Intensive Care Units (NICUs) routinely use point‐of‐care ultrasound in their clinical practice with cranial and cardiac ultrasounds as standard of care [14]. Interest in expanding point‐of‐care ultrasound to include lung and abdomen is considerable.

The combined diagnostic use of point‐of‐care abdominal ultrasound for bowel assessment and X‐ray to support the diagnosis of NEC is feasible [15]. The optimal management of NEC requires repeated X‐rays for close monitoring of the disease progression. The advantages of adding bowel ultrasound assessments include direct visualisation of the bowel wall, bowel peristalsis, vascularity and detection of intra‐abdominal fluid collections, allowing for clinical decision‐making on disease severity guiding management [8, 9, 11]. Furthermore, the high specificity of bowel ultrasound findings in NEC is of value, as the absence of ultrasound findings can make the diagnosis of NEC less likely and provide reassurance to clinicians [16].

This survey is an important step towards understanding the interest in point‐of‐care bowel ultrasound among neonatal clinicians in Australia, New Zealand and Singapore. It explores the barriers and facilitators in bringing point‐of‐care bowel ultrasound as a diagnostic tool within the reach and choice of neonatal clinicians. By identifying these interests, the survey aimed to facilitate the implementation and skill learning required to enhance its clinical use and improve the diagnosis of NEC, potentially leading to better patient outcomes.

## 2 Methods

### 2.1 Survey Design

This is a quantitative study using a cross‐sectional, online, single‐invitation survey, undertaken to assess the current knowledge and interest among neonatal clinicians using point‐of‐care ultrasound at NICUs across Australia, New Zealand and Singapore. The focus of the survey was to investigate their views and opinions on using point‐of‐care bowel ultrasound for the diagnosis of NEC.

The participating tertiary NICU Level III nurseries were identified by the ANZNN (Australian and New Zealand Neonatal Network), and the NICU directors were contacted via email with an invitation to participate. A questionnaire was developed using Research Electronic Data Capture (REDCap, Vanderbilt University, Nashville, TN, USA). A unique link to access the questionnaire along with the request‐to‐participate introductory letter was distributed. This survey was open for two weeks between 15 November 2023 and 1 December 2023, with no reminders sent. Participation in this survey was voluntary, and participants consented by their choice to click on the request link. All questions were single or multiple‐choice, with a single free‐text question for any additional comments at the end of the survey. The survey (in Appendix S1) requested information about a unit's practice of point‐of‐care abdominal ultrasound.

### 2.2 Data Analysis

Survey data were analysed using descriptive statistical analyses, and the proportions and percentages of the responses were reported.

## 3 Results

65 individual responses, from 29 NICUs across the ANZNN were received.

Among the participating neonatal clinicians, 48% were fully certified by the Australian Society of Ultrasound in Medicine (ASUM) after completing the Certificate in Clinician‐Performed Ultrasound (CCPU) course and 60% had more than five years of point‐of‐care ultrasound practice experience postcertification.

All tertiary units across Australia and New Zealand and one unit from Singapore (as an ANZNN participating unit) were represented in the survey. The units provided perinatal services (37%), surgical services (17%) or both perinatal and surgical services (46%). All reported access to point‐of‐care ultrasound machines, with 24‐h availability. A majority (67%) reported access to the high‐frequency linear transducers (5–18 Hz) required for ideal imaging of the neonatal bowel **(**Table 1 **)**.

**TABLE 1:** Demographics of the respondents to the survey.

Participants (*n* = 65)	Number (%)
Participating units	29/29
Certificate in clinician‐performed ultrasound (CCPU)	
CCPU certified	31 (48%)
CCPU in training	28 (43%)
CCPU training completed and awaiting certification	6 (9%)
Availability of resources	
24‐h access to ultrasound machine	65 (100%)
24‐h access to surgical consultation within the hospital	52 (78%)
Awareness on the published literature on point‐of‐care bowel ultrasound in the diagnosis of NEC	
Aware	55 (85%)
Not sure	10 (15%)

## 4 Opinion of Surveyed Neonatal Clinicians

In this study, 87% of the participants agreed that distinguishing early NEC from other causes of abdominal distention in a haemodynamically unstable preterm infant was a common clinical issue. Most (82%) of these clinicians would consider performing an ultrasound of the bowel in cases with suspected NEC.

**FIGURE 1:**
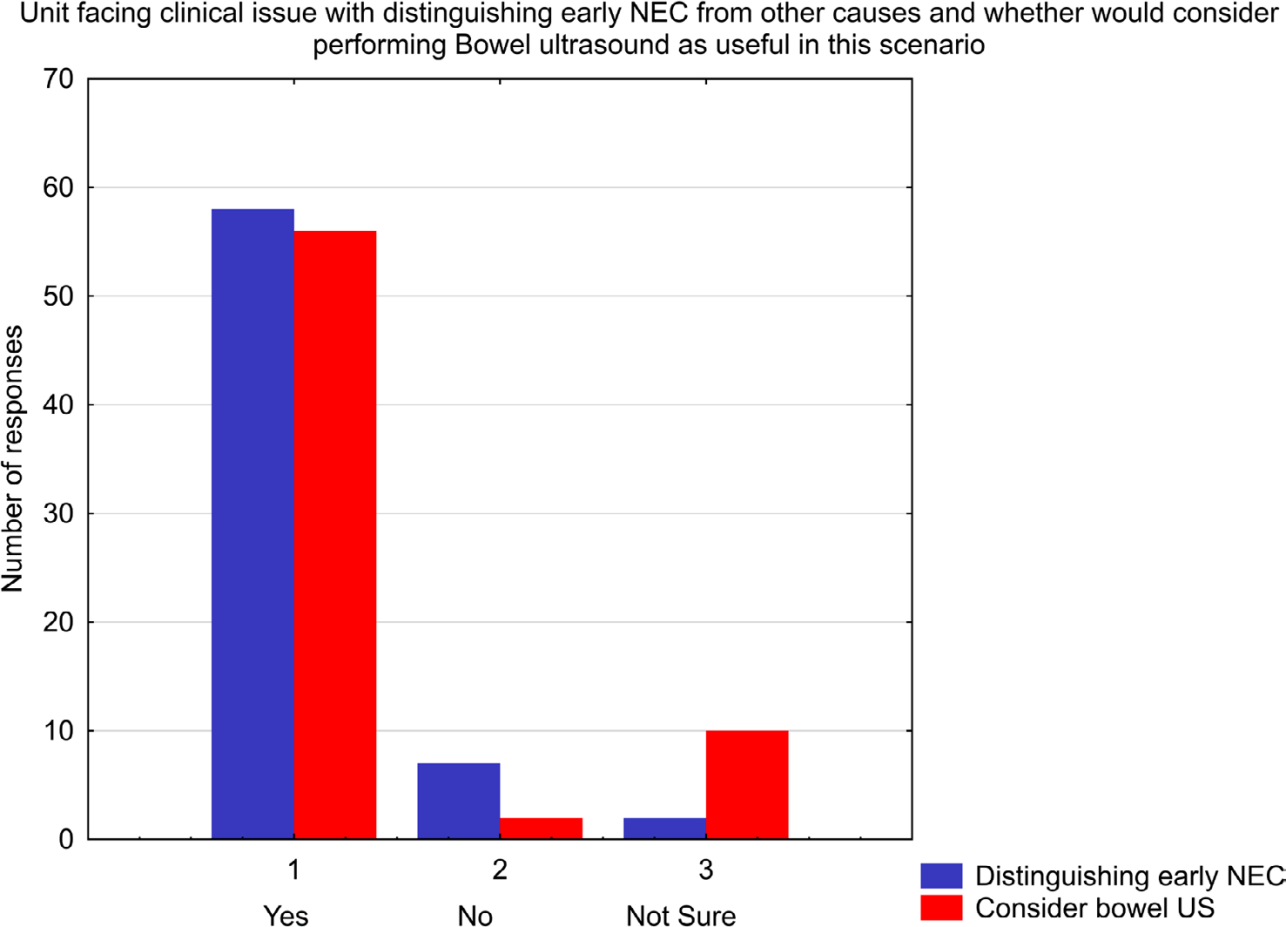
Responses for the questions: Is distinguishing early necrotising enterocolitis from other causes of abdominal distention in a haemodynamically unstable preterm infant a common issue in your unit? Would performing a bowel ultrasound to help with diagnostic assessment in the above scenario be helpful?

While more than half of the participants agreed that performing a point‐of‐care abdominal bowel ultrasound in suspected NEC was not standard of care, currently 46% of the units had access to radiology services to request a bowel ultrasound examination. In cases with possible NEC, 45% would consider a paediatric radiologist or sonographer consultation from paediatric radiology services to request a bowel ultrasound (Figure 1).

### 4.1 Training Programme

**FIGURE 2:**
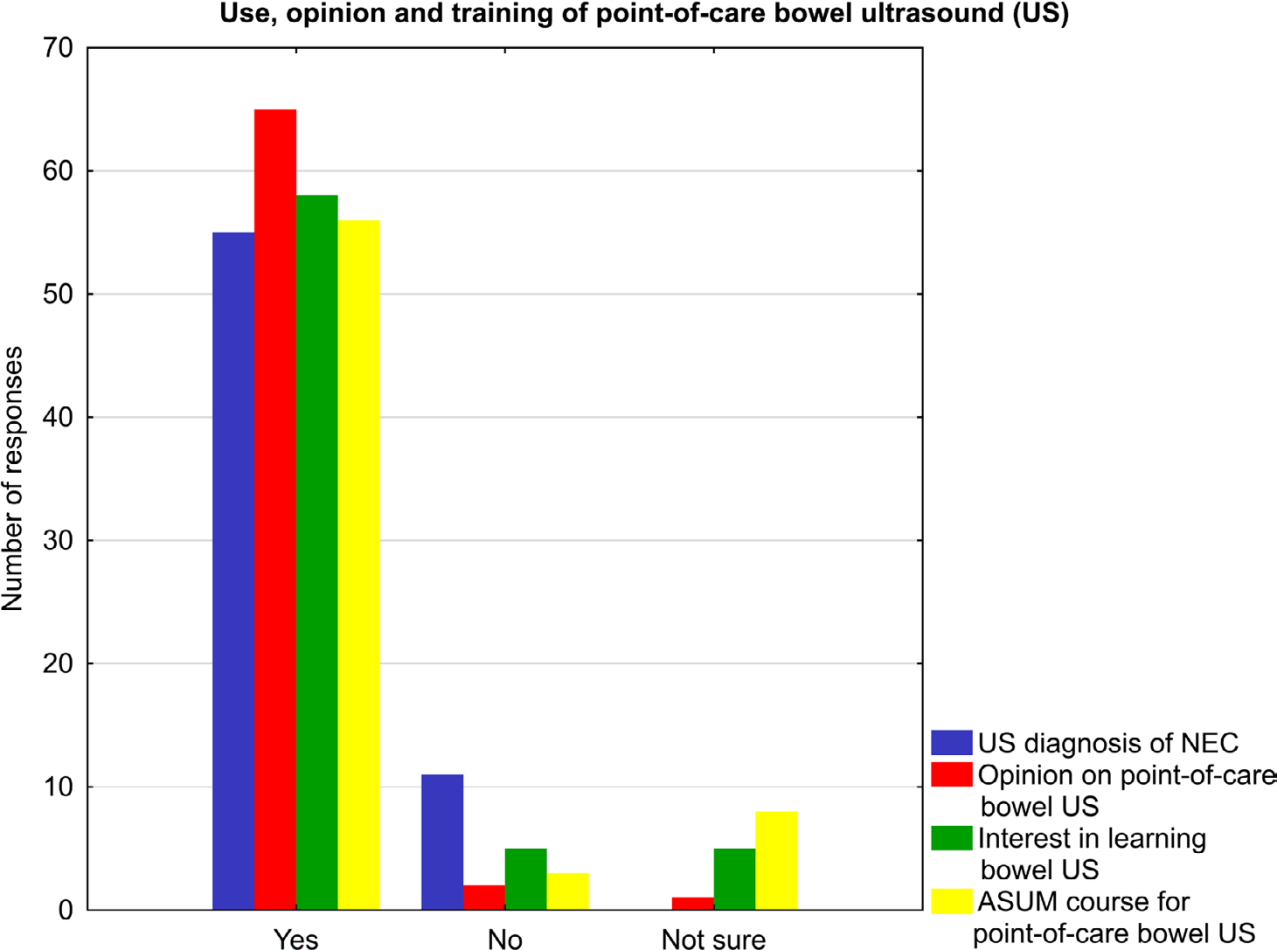
Responses for the questions: Are you aware of studies on the utility of bowel ultrasound in diagnosing NEC? What is your opinion on the clinical utility of point‐of‐care bowel ultrasound in addition to plain radiography and clinical assessment? Would you be interested in a training course on performing point‐of‐care abdominal ultrasound to assess the bowel?

Almost all (95%) of the respondents agreed that performing a point‐of‐care bowel ultrasound in diagnosing NEC is helpful or may be helpful, and 87% were interested in a training course with ASUM. Less than 5% of the participants were of the opinion that bowel ultrasound in NEC was of no clinical importance (Figure 2).

## 5 Discussion

This study involved neonatal clinicians practising point‐of‐care ultrasound in 29 tertiary neonatal units across Australia, New Zealand and Singapore, and explored clinical interest in the adoption and utility of performing point‐of‐care bowel ultrasound in the diagnosis of NEC. This study included opinions from neonatal clinicians fully certified (CCPU) from ASUM and those in training. There is currently no dedicated point‐of‐care abdominal ultrasound training for bowel assessment within Australasia. Most (95%) of the participating clinicians were interested in exploring the utility of point‐of‐care bowel ultrasound for the diagnosis of NEC, with 87% willing to commit to undergo training. This survey has allowed identification of neonatal units with a strong interest and experience in this field, opening new opportunities for collaboration.

Access to high‐frequency (5–18 Hz) point‐of‐care ultrasound machines around the clock was not identified as an issue, with at least half of the neonatal clinicians reporting access to radiology services should a bowel ultrasound be requested.

While the survey responses facilitate a review of the feasibility of expanding the scope of this skill, our study is limited by the number of participants and the voluntary nature, suggesting self‐selection of participants with greater interest in this topic. Our survey did not include distribution within other relevant staff such as paediatric radiologists, paediatric surgeons or nursing staff, which could potentially influence the process, timing and perceived clinical benefit of ultrasound in cases with NEC.

The overall incidence of NEC in Australasia is low, (Data from the Australian and New Zealand Neonatal Network‐ANZNN) and a major challenge is the unpredictable availability of a case required for hands‐on training. Accurate identification and diagnosis of pathological bowel features in neonates by ultrasound is a steep learning curve requiring extensive hands‐on experience. To date, data are lacking on the normal bowel wall thickness measurements in extremely premature infants, of interest to neonatal clinicians managing advancing ages of prematurity. Hence, although the state‐of‐the‐art findings in NEC and bowel ultrasound were described almost two decades ago [8, 9], there has been a delay in the adaptability of this skill by neonatal clinicians. Barriers to the routine use of bowel ultrasound in the diagnosis of NEC include lack of education and training opportunities for sonographers and radiologists, low NEC case numbers and clinician unfamiliarity with the use of the abdominal ultrasound information for clinical diagnosis.

Despite these challenges, real‐time visualisation of the bowel is valuable. Optimal management in NEC requires repeated abdominal screening routinely done by X‐rays in current clinical practice. The transition from medical to surgical care represents a critical decision‐making point in NEC management. The abdominal ultrasound findings in NEC most strongly associated with surgery or death are free air, absent peristalsis, complex ascites and focal fluid collection [16]. The detection of portal venous gas and pneumatosis intestinalis by ultrasound, particularly if these signs are not seen on the plain radiograph, is extremely helpful for the accurate and prompt diagnosis of NEC. This justifies the need for increased access to ultrasound services and/or evolution of neonatal clinician performed bowel ultrasound. On the other hand, the absence of any bowel pathology on ultrasound offers reassurance to clinicians managing extremely preterm infants with abdominal distention, on noninvasive respiratory support presenting with feed intolerance. Thus, there is clinical value in integrating X‐ray and ultrasound for a comprehensive assessment of the bowel in cases with suspected NEC.

Our study provides a foundation for a collaborative approach in developing, learning and sharing knowledge and interpretation of point‐of‐care abdominal ultrasound and bowel ultrasound findings in diagnosing NEC. This initiative, spanning across ANZNN tertiary neonatal units, allows us to advance in this emerging field that has an evolving surge in interest globally. The potential impact of these findings on clinical practice advances the future of bowel ultrasound skills in the hands of neonatal clinicians.

## Author Contributions

A.P. (Archana Priyadarshi) created the design of the survey. S.R., N.B., and M.T. provided the review of the content. S.M. and A.P. (Amy Phu) contributed to the data collection. All authors agree with the content of the submitted manuscript.

## Ethics Statement

Ethics approval was obtained from The Sydney Children's Hospital Ethical Network (HREC reference number 2019/ETH10657).

## Conflicts of Interest

The authors declare no conflicts of interest.

## Supporting information


**Appendix S1.** Survey questions.
