# Supplementary material for: An Australasian Survey of Neonatal Clinicians on Clinical Utility of Point‐of‐Care Bowel Ultrasound in Diagnosis of Necrotising Enterocolitis
Source: Australas J Ultrasound Med. 2025 Jun 13;28(3):e70007. doi: 10.1002/ajum.70007 (PMC12163532; doi:10.1002/ajum.70007)
Supplement: Supplementary file 1 — Appendix S1. Survey questions. [file AJUM-28-0-s001.docx]

Appendix: Survey questions

1. **Please specify your CCPU status, please tick:**

In training

Certified

1. **If certified, please specify the number of years past certification (range in years), please tick:**

0-3

3-5

>5

1. **Please name your unit, hospital, location**

…………………………………………………

1. **Is your neonatal unit**

Perinatal unit

Surgical unit

Both perinatal and surgical unit

1. **Please state the number of beds in your neonatal unit**

..........................................................................................

1. **At this stage is distinguishing Early Necrotising Enterocolitis from other causes of abdominal distention in a haemodynamically unstable preterm infant**

A common issue for your unit? Yes / No / Undecided

Would you consider asking for BUS to help diagnostic assessment?

Yes / No / Undecided

1. **Does your unit have access to Paediatric Radiology consultations 24 hours on all days?**

Yes

No

1. **In the case of possible Necrotising Enterocolitis do you consider asking a Paediatric radiologist or sonographer from Paediatric Radiology to perform BUS?**

Yes

No

Undecided

If no, are you aware of studies on utility of bowel ultrasound in the diagnosis of NEC?

................................................................................

1. **What is your opinion on the clinical utility of point-of care bowel ultrasound in addition to plain radiography and clinical assessment?**

Useful

May be useful

Not useful

Don`t know

1. **Would you be interested in training course on learning how to perform bowel ultrasound?**

Yes

No

Undecided

1. **Regarding the Ultrasound machine, please provide information on:**

Brand ……………………..

Model………………………

Do you have a linear probe ……yes/no?

If you do-What Hz (frequency) is your linear probe…….

1. **Do you think ASUM should explore the feasibility of bowel ultrasound course for CCPU point of care neonatal clinicians?**

Yes

No

Not sure

1. **Does your neonatal unit have access to paediatric surgical opinion within the hospital 24 hours a day for all days?**

Yes

No - transfer to surgical unit via retrieval team

- Intra-hospital medical staff escort for transfer to surgical unit
